# A nomogram model to predict the high risk of lower live birth probability in young women undergoing the first IVF-ET cycle: a retrospective study

**DOI:** 10.3389/fendo.2024.1401385

**Published:** 2024-12-19

**Authors:** Chang Liu, Peipei Pan, Beihai Li, Yili Teng

**Affiliations:** ^1^ Reproductive Medicine Center, The First Affiliated Hospital of Wenzhou Medical University, Wenzhou, Zhejiang, China; ^2^ Department of Obstetrics and Gynecology, Yueqing Fifth People’s Hospital, Wenzhou, Zhejiang, China

**Keywords:** diminished ovarian reserve, live birth, ovum pick-up, prediction nomogram, IVF/ICSI

## Abstract

**Objective:**

To build a prediction nomogram for early prediction of live birth probabilities according to number of oocytes retrieved in women ≤ 35 years of age.

**Methods:**

A prediction model was built including 9265 infertile women ≤ 35 years of age accepting their first ovum pick-up cycle from January 2018 to December 2022. Least absolute shrinkage and selection operator (LASSO) regression was performed to identify independent predictors and establish a nomogram to predict reproductive outcomes. Both discrimination and calibration were assessed by bootstrapping with 1000 resamples.

**Results:**

The critical threshold for the number of retrieved oocytes associated with cumulative live birth was determined as 10.5 (AUC: 0.824). Consequently, a nomogram was constructed to predict the likelihood of obtaining fewer than 10 oocytes at one oocyte retrieval cycle. There were five indicators significantly related to the risk of obtaining less than 10 oocytes at one oocyte retrieval cycle, including age, antral follicle count (AFC), anti-Mullerian hormone (AMH), follicle-stimulating hormone (FSH), and FSH to luteinizing hormone ratio. These factors were subsequently used to develop a nomogram prediction model. The model’s performance was evaluated using the area under the curve (AUC), concordance index (C-index), and calibration curves, which indicated fair predictive ability and good calibration.

**Conclusion:**

We developed and validated a nomogram based on five ovarian reserve indicators to predict the risk of retrieving fewer than 10 oocytes at one oocyte retrieval cycle in women ≤ 35 years of age. The model demonstrated good discrimination and calibration, indicating its reliability for clinical application. This nomogram offers a practical and accurate tool for early identification of young women with potentially decreased ovarian reserve, enabling timely intervention and personalized management strategies.

## Introduction

To date, more than 10 million babies have been born worldwide through *in vitro* fertilization (IVF) (1). Female fertility is a critical aspect of reproductive health, intricately linked to ovarian reserve—the quantity and quality of remaining eggs in a woman’s ovaries. Studies indicate a significant decline in women’s fertility after the age of 35 (2, 3). Consequently, women aged ≤ 35 undergoing IVF typically experience more favorable reproductive outcomes compared to older individuals.

Diminished ovarian reserve (DOR) is a condition characterized by a reduced quantity and/or quality of oocytes, which can lead to fertilization failure, embryonic aneuploidy, and increased miscarriage rates, significantly affecting live birth outcomes. Although commonly associated with advanced maternal age, DOR is increasingly observed in younger women due to factors such as ovarian surgery, unhealthy lifestyles, high-pressure work conditions, environmental pollution, and genetic predispositions (4). This shift highlights the need for personalized fertility management strategies in this younger demographic.

Well-established ovarian reserve indicators include serum follicle-stimulating hormone (FSH), the FSH to luteinizing hormone ratio, anti-Mullerian hormone (AMH) levels and antral follicle counts (AFC). These markers are routinely used in clinical practice to assess ovarian function and guide IVF protocols. The early hormonal manifestations of DOR often involve elevated FSH levels (>10 U/L) and decreased bilateral AFC during early follicular phase. Meta-analysis have shown that although serum AMH levels were significantly higher in women with live birth, low AMH levels should not be the sole criterion for rejecting IVF treatment, especially among young women (5). Therefore, these indicators alone are insufficient for determining optimal oocyte retrieval strategies to maximize live birth outcomes, especially in younger women with DOR.

Predicting live birth outcomes in women with DOR remains challenging, particularly in those aged ≤ 35 years who generally have better reproductive outcomes. Despite diminished ovarian reserve, younger women with DOR still have reasonable chances of achieving a pregnancy, although their prognosis is influenced by the number of oocytes retrieved per oocyte retrieval cycle (6, 7). Clinicians often face a critical decision point in this group: determining whether to proceed with embryo transfer after a single ovarian stimulation cycle or to recommend additional stimulation cycles to retrieve more oocytes. Current guidelines lack specific recommendations for this scenario, leading to variability in clinical practice.

Therefore, understanding the predictive indicators of reproductive outcomes in this specific age group is pivotal for proactive fertility management and personalized interventions. To this end, this retrospective study aims to establish a user-friendly nomogram that incorporates commonly used clinical parameters to predict live birth probabilities in women ≤ 35 years of age undergoing IVF.

## Patients and methods

### Study design and population

This retrospective study was conducted among women ≤ 35 years old who underwent their first IVF/ICSI cycle at the Reproductive Center of the First Affiliated Hospital of Wenzhou Medical University between January 2018 and December 2022. The inclusion criteria were as follows: (1) data for only the first IVF or ICSI cycle included, (2) couples with normal karyotype results confirmed by chromosomal analysis; (3) sperm sample collected by masturbation. Patients were excluded if they had (1) abnormal karyotype results in either partner, (2) previous history of IVF or ICSI treatment, (3) incomplete baseline data. The study protocol was approved by the Ethics Committees of the First Affiliated Hospital of Wenzhou Medical University (protocol number: KY2023-R224) with a waiver for informed consent.

### Ovarian stimulation and embryo transfer procedures

All patients received a standardized ovarian stimulation protocol, oocyte retrieval, fertilization, and embryo transfer, which were specifically described in previous study (8). The oocyte maturation was triggered using either human chorionic gonadotropin, leuprolide acetate, or a combination of both. Oocyte retrieval was performed 36 hours after the trigger. Before embryo transfers, patients were instructed to follow a standardized protocol and received luteal support by either vaginal or intramuscular progesterone supplementation. No more than two embryos were transferred in either fresh or frozen-thawed embryo transfer cycle.

### Development of model for predicting CLB according to the number of retrieved oocytes

Cumulative live birth was defined as at least one live birth resulting from one oocyte retrieval cycle in either fresh embryo transfer or subsequent frozen embryo transfer in relation to the number of oocytes retrieved (9). Good-quality embryos on Day 3 were defined as those graded as 7A, 7B, 8A, 8B, 9A or 9B. Good-quality embryos on Day 5 or Day 6 were defined as those graded over 3BB. Recognizing the crucial role of embryo quality in achieving live births, we initially determined the cutoff value for the number of good-quality embryos predictive of CLB using data from 7379 cycles with complete follow-up records. Complete follow-up records were defined as: 1) patients achieved a live birth after transferring embryos from the first oocyte retrieval cycle; 2) patients failed to conceive after embryo transfer, with no remaining embryos from the first oocyte retrieval cycle. Incomplete follow-up records were defined as: 1) patients discontinued treatment without achieving a live birth but had remaining embryos from the first oocyte retrieval cycle; 2) patients got pregnant after embryo transfer but had not yet achieved a live birth; 3) patients were lost to follow-up. The flowchart of the data selection was presented in **Supplementary Figure 1**. The preliminary receiver operating characteristic (ROC) curve analysis indicated a cutoff value of 2.5 (AUC: 0.723) (**Supplementary Figure 2A**). Subsequently, we classified the number of good-quality embryos as ≤ 2 or > 2, with a cutoff value for the number of retrieved oocytes determined as 10.5 (AUC: 0.824) (**Supplementary Figure 2B**). Therefore, we constructed a nomogram to predict the risk of obtaining fewer than 10 oocytes at one oocyte retrieval cycle utilizing data from a total of 9265 cycles.

### Outcome of interest

The primary outcome of interest was the risk of retrieving fewer than 10 oocytes at one oocyte retrieval cycle in infertile patients ≤ 35 years of age, aiming to provide clinicians with a practical reference for decision-making on whether to proceed with additional oocyte retrieval cycle or proceed to embryo transfer. Potential influencing factors include demographic characteristics and ovarian reserve indicators, as we focused on incorporating commonly used and essential clinical variables to develop a simple and practical prediction model. To address possible biases, we carefully controlled for selection bias by clearly defining inclusion and exclusion criteria. Information bias was minimized through standardized procedures for hormone measurements, AFC assessments, and laboratory practices, while confounding bias was addressed using LASSO regression analysis. A comprehensive dataset was compiled by extracting relevant clinical information from electronic medical records.

### Statistical analysis

All statistical analyses were conducted using R version 4.3.2 software with rms, pROC, ggplot2 and ggDCA packages. The data were randomly divided into a training set (70%) and a verification set (30%) in a 7:3 ratio. The training set was utilized for model training, while the verification set was employed for model verification. Continuous variables were presented as mean ± standard deviation for normally distributed data, with comparison between groups performed using independent sample t-tests. Skewed continuous data were expressed as median (M) and interquartile range (IQR: P25-P75), with between-group comparisons conducted using the Kruskal-Wallis test. Count data were expressed as percentages (%), with group comparisons performed using the chi-square test, supplemented by Fisher’s exact probability test when the chi-square test was deemed inappropriate.

The predictive model is constructed based on the training set. Initially, potential predictors of outcome events are screened out based on univariate logistic regression analysis (P<0.05). Furthermore, the least absolute shrinkage and selection operator (LASSO) regression algorithm is employed to select significant characteristics, where non-zero coefficients indicate significance. The optimal parameter configuration was determined through 10-fold cross-validation. Coefficients were determined based on the lambda value (1se) of a standard error corresponding to the minimum deviation of the distance, by which the variables with non-zero coefficients were screened. Multivariate logistic regression analysis (stepwise, two-way) was then conducted based on the selected variables. The prediction model nomogram was constructed using variables deemed statistically significant in the stepwise method. Verification of the constructed nomogram was performed using 1000 bootstrap samples. Calibration curves were generated to evaluate model calibration. Hosmer-Lemeshow test was performed to evaluate the goodness of fit of the model. Additionally, through ROC curve analysis, AUC, sensitivity, specificity and other indicators were calculated to evaluate the efficacy of the nomogram, and clinical decision curve analysis (DCA) was constructed to evaluate the clinical application value of the model and quantify the net benefit within the threshold probability range. Finally, the constructed model underwent verification in the validation set. All statistical testes were two-sided, with *P*< 0.05 considered statistically significant.

## Results

### Baseline characteristics

A total of 9,265 patients, including 6,485 in the training group and 2,780 in the validation group, were enrolled in this study. **Table 1** presented the baseline characteristics of the groups. The results showed that there were no significant differences in age, duration of infertility, body mass index (BMI), antral follicle count (AFC), anti-Mullerian hormone (AMH) levels, basal follicle-stimulating hormone (FSH), basal estradiol (E2), FSH/LH ratio, or duration of menstrual cycle between the groups.

**Table 1:** Basis characteristics of training group and validation group.

Variables	Training (n = 6485)	Validation (n = 2780)	*P* value
Age (year) (Median, IQR)	30.00 (28.00, 32.00)	30.00 (28.00, 33.00)	0.26
Infertility duration, (year) (Median, IQR)	3.00 (2.00, 4.00)	3.00 (2.00, 5.00)	0.12
BMI (kg/m^2^) (Median, IQR)	21.48 (19.63, 24.03)	21.48 (19.60, 24.03)	0.88
AFC (n) (Median, IQR)	17.00 (12.00, 24.00)	17.00 (12.00, 24.00)	0.95
AMH (ng/ml) (Median, IQR)	4.07 (2.38, 6.59)	4.01 (2.35, 6.71)	0.84
Basal FSH (IU/L) (Median, IQR)	7.49 (6.32, 9.01)	7.50 (6.29, 9.05)	0.92
FSH/LH ratio (Median, IQR)	1.54 (1.11, 2.22)	1.55 (1.11, 2.21)	0.78
Basal E2 (pmol/L) (Median, IQR)	162.00 (118.00, 216.00)	165.00 (118.60, 220.62)	0.25
Duration of menstrual cycle (day) (Median, IQR)	28.00 (28.00, 30.00)	28.00 (28.00, 30.00)	0.44

BMI, body mass index; AFC, antral follicle count; AMH, anti-Mullerian hormone; FSH, follicle-stimulating hormone; FSH/LH ratio, follicle-stimulating hormone to luteinizing hormone ratio; E2, estradiol.

### Screening for predictive factors

The univariate associations of the potential predictors for CLB were presented in **Table 2**. Eight statistically significant predictors were identified, namely age, duration of infertility, AFC, AMH, basal FSH, basal E2, FSH/LH ratio, and the duration of menstrual cycle.

**Table 2:** Univariate and multivariate logistic analysis of factors predicting the risk of fewer than 10 oocytes retrieved at one oocyte retrieval cycle in the training group.

variable	Univariate logistic analysis		Multivariate logistic analysis	
	OR (95%CI)	*P* value	OR (95%CI)	*P* value
Age	1.13 (1.11, 1.15)	<0.001	1.05 (1.03, 1.07)	<0.001
Infertility duration	1.04 (1.02, 1.06)	0.001	/	/
BMI	1.01 (0.99, 1.03)	0.11	/	/
AFC	0.87 (0.86, 0.87)	<0.001	0.92 (0.91, 0.93)	<0.001
AMH	0.67 (0.65, 0.69)	<0.001	0.85 (0.82, 0.88)	<0.001
Basal FSH	1.29 (1.26, 1.32)	<0.001	1.10 (1.10,1.13)	<0.001
FSH/LH ratio	2.18 (2.05, 2.32)	<0.001	1.22 (1.13,1.32)	<0.001
Basal E2	1.00 (1.00, 1.00)	<0.001	/	/
Duration of menstrual cycle	0.95 (0.95, 0.96)	<0.001	/	/

BMI, body mass index; AFC, antral follicle count; AMH, anti-Mullerian hormone; FSH, follicle-stimulating hormone; FSH/LH ratio, follicle-stimulating hormone to luteinizing hormone ratio; E2, estradiol.

**Figure 1:**
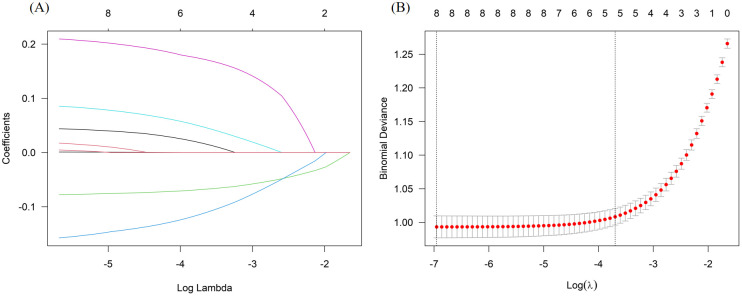
LASSO regression **(A)** and cross-validation figure **(B)**.

LASSO regression analysis was conducted using the aforementioned eight independent variables (**Figure 1A**). Baseline covariates with non-zero regression coefficients, specifically age, AFC, AMH, FSH, and FSH/LH, were retained. Cross-validation revealed that as λ increases, the model incorporates fewer baseline covariates (**Figure 1B**). Lambda.lse (0.02480917) indicated a model with good performance but the lowest number of independent variables.

Multivariate logistic regression analysis identified five independent predictors for retrieving fewer than 10 oocytes at one oocyte retrieval cycle, as follows: age (odds ratio [OR] 1.05, 95% confidence interval [CI] 1.03-1.07), AFC (OR 0.92, 95% CI 0.91-0.93), AMH (OR 0.85, 95% CI 0.82-0.88), FSH (OR 1.10, 95% CI 1.07-1.13), FSH/LH (OR 1.22, 95% CI 1.13-1.32) (**Table 2**).

### Risk prediction nomogram development

**Figure 2:**
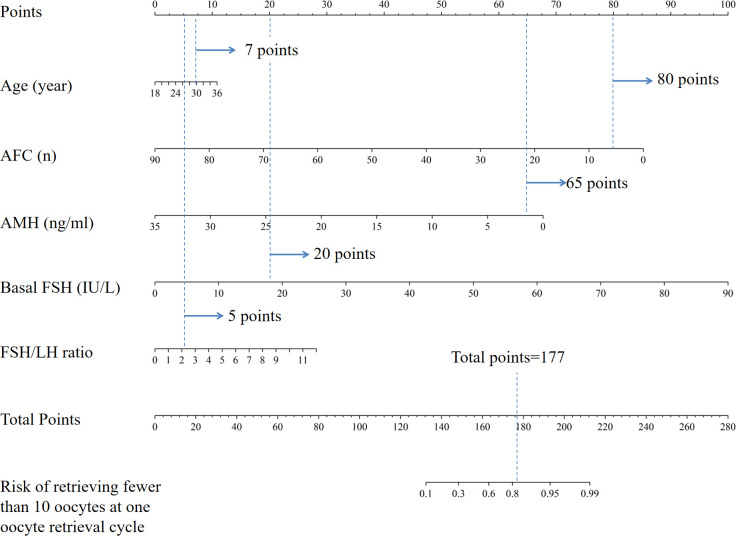
Nomogram for the risk of retrieving fewer than 10 oocytes at one oocyte retrieval cycle. AFC, antral follicle account; AMH, anti-Mullerian hormone; FSH, follicle stimulating hormone; FSH/LH ratio, follicle-stimulating hormone to luteinizing hormone ratio.

The logistic regression model was constructed using the aforementioned five indicators, which were subsequently integrated into the nomogram (**Figure 2**). By adding up the score of each item, the sum of the points is recorded as the total score. After tracing the total score downward to the horizontal axis, the predicted likelihood of retrieving fewer than 10 oocytes at one oocyte retrieval cycle was determined. Higher total points for each patient corresponded to a higher likelihood of retrieving fewer than 10 oocytes at one oocyte retrieval cycle. For example, for a 30-year-old woman (7 points) with antral follicle counts of 6 (80 points), AMH of 2.0 ng/ml (65 points), basal FSH of 18 IU/L (20 points), and FSH/LH ratio of 2.2 (5 points), the total score adds up to 177, which corresponds to an approximate probability of 82% for retrieving fewer than 10 oocytes at one oocyte retrieval cycle. Therefore, this woman’s risk of retrieving fewer than 10 oocytes is 82%, and it may be appropriate to consider a strategy of continued oocyte retrieval.

We have also constructed an simple online dynamic nomogram website, available at https://edopor.shinyapps.io/DynNomapp/. By inputting numerical value of each variables, the prediction value could be calculated immediately. For example, the usage of the nomogram is illustrated with an assumptive woman as: 30 years old, AFC=7, AMH=1.0ng/ml, FSH=10U/L, FSH/LH ratio=2. The total score added up to 164 for this woman, which represents approximately 0.68 of probability of retrieving fewer than 10 oocytes at one oocyte retrieval cycle (**Supplementary Figure 3**). However, a limitation of this website is that the numerical values cannot be entered with perfect precision, and it only provides a range for each variable. As a result, the prediction of the probability of retrieving fewer than 10 oocytes can only be an approximate estimate. The website is still under development and will be further improved to allow for more accurate predictions.

### Predictive accuracy and net benefit of the nomogram

**Figure 3:**
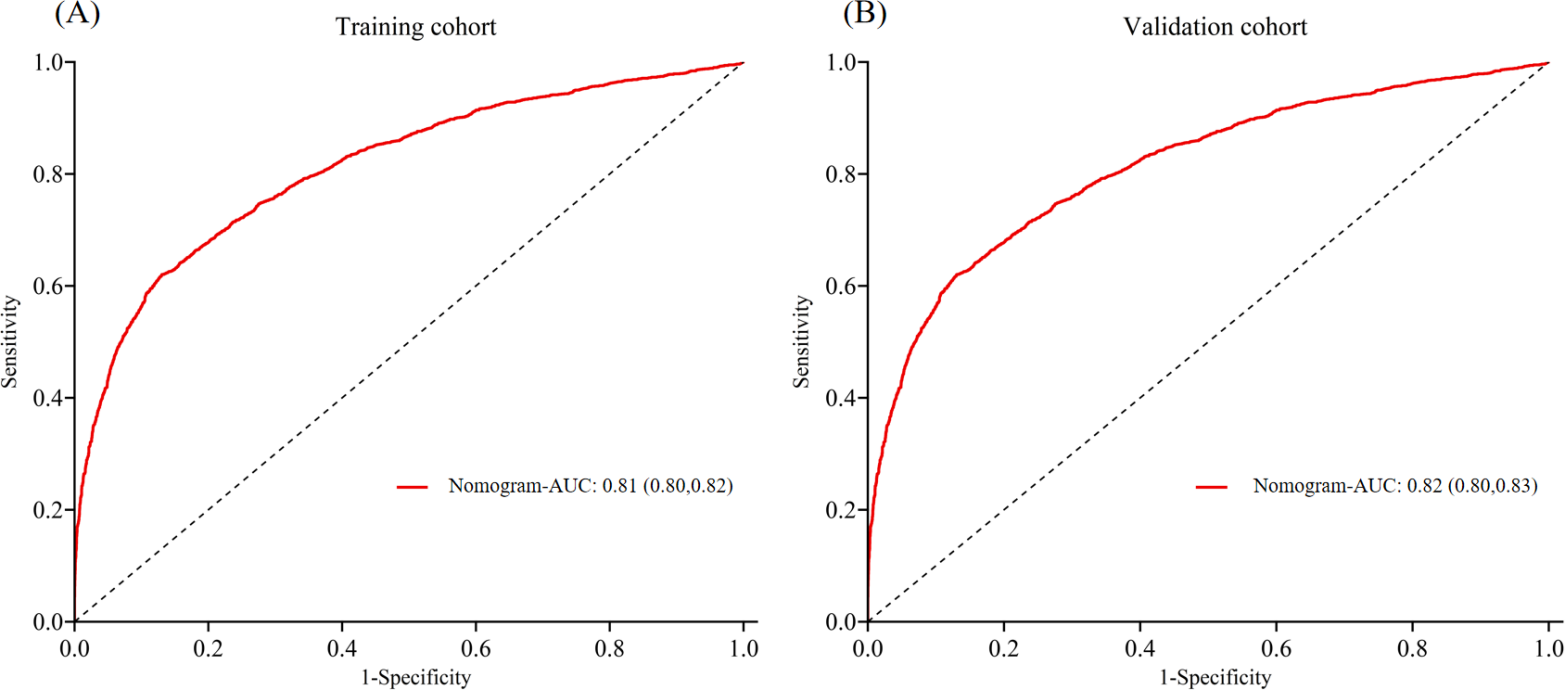
ROC curves of **(A)** Training cohort and **(B)** Validation cohort. ROC, receiver operating characteristic; AUC, area under the ROC curve.

**Figure 4:**
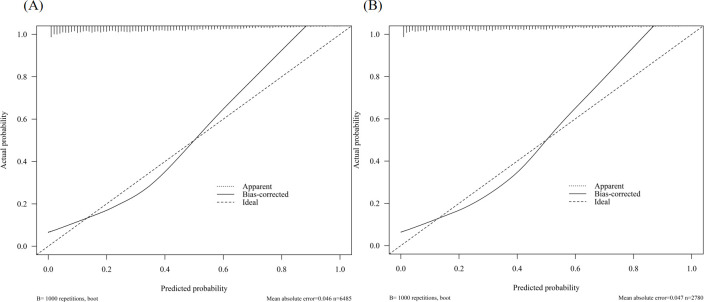
Calibration curve for predicting the probability of retrieving fewer than 10 oocytes at one oocyte retrieval cycle: **(A)** Training cohort; **(B)** Validation cohort.

**Figure 5:**
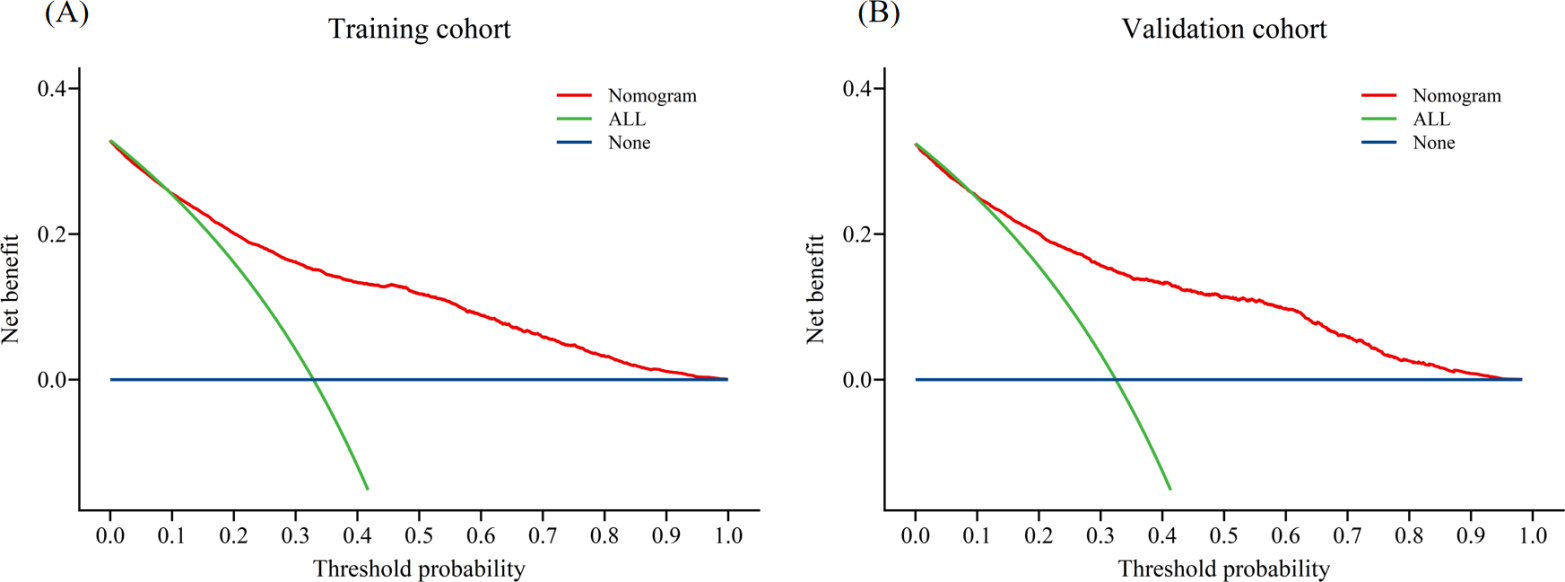
Decision curve analysis in prediction of fewer than 10 oocytes retrieved at one oocyte retrieval cycle: **(A)** Training cohort; **(B)** Validation cohort.

In the training cohort, the AUC was 0.81 (**Figure 3A**), and the calibration curve was close to the ideal diagonal line (**Figure 4A**). Furthermore, the DCA demonstrated significantly better net benefit in the predictive model (**Figure 5A**).

In addition, 2,780 patients were included in the external validation to assess the nomogram. The AUC was 0.82 (**Figure 3B**), indicating good accuracy of the nomogram. Meanwhile, the model exhibited good consistency, with the calibration curve of the validation cohort closely resembling the ideal diagonal line (**Figure 4B**). Moreover, DCA showed significant net benefit of the predictive model, consistent with the findings in the validation cohort (**Figure 5B**). These data underscore the significant potential of our nomogram for clinical decision-making. The cut-off value of the model was 0.46 and 0.41 in training and validation groups, respectively. At these cut-off points, the sensitivity was 62.0% and the specificity was 87.0% in the training group, while in the validation group, the sensitivity was 67.7% and the specificity was 81.5%. The C-index of the predictive model was 0.81 (95% CI: 0.80-0.82) in the training group and 0.82 (95% CI: 0.80-0.83) in the validation group (**Table 3**). These results indicated that the model exhibited fair performance. For example, if a patient has a predicted probability of 70% from the nomogram (indicating a 70% likelihood of retrieving fewer than 10 oocytes in one cycle), the clinician can confidently use the model to guide the decision, such as recommending additional oocyte retrieval. Conversely, if the predicted probability is 10%, the DCA graph suggests adopting embryo transfer (blue line) strategy, as it provides a higher net benefit.

**Table 3:** ROC analysis of the nomogram in the training group and validation group.

Outcome	Model	Training group				Validation group			
		Cut-off value	Youden index	AUC	C-index	Cut-off value	Youden index	AUC	C-index
Retrieving less than 10 oocytes at one oocyte retrieval cycle	Age + AFC + AMH + FSH + FSH/LH ratio	0.46	0.49	0.81	0.81 (0.80, 0.82)	0.41	0.49	0.82	0.82 (0.80, 0.83)

ROC, receiver operating characteristic; AFC, antral follicle account; AMH, anti-Mullerian hormone; FSH, follicle stimulating hormone; FSH/LH ratio, follicle-stimulating hormone to luteinizing hormone ratio; AUC, area under the curve.

## Discussion

Our study is the first to develop a model for predicting cumulative live birth according to the risk of retrieving fewer than 10 oocytes at one oocyte retrieval cycle in infertile patients ≤35 years of age. The nomogram integrates several factors including age, AFC, AMH, FSH, and FSH/LH ratio, all of which are easily obtainable before commencing IVF treatment.

Ovarian reserve refers to both the quantity and quality of follicles in the ovaries, which can reflect the potential of follicles to develop into good-quality oocytes, thus representing the reproductive potential of women (10). Women with DOR often experience low ovarian response and retrieve fewer oocytes during ovarian stimulation, resulting in a reduced likelihood of live birth compared to those with normal ovarian reserve. Enhancing the live birth rate hinges on obtaining more mature oocytes during controlled ovarian hyperstimulation to yield a greater number of transferable and high-quality embryos. In recent years, a trend has emerged where women with DOR are younger, significantly impacting their reproductive function and quality of life. However, a study conducted in women <40 years age indicated that once an euploid embryo was transferred, its developmental potential to achieve a live birth would not be impacted by the DOR diagnosis (11). Although age is primarily associated with the rapid decline in the production of healthy and high-quality oocytes in women over 35 years of age, women with DOR have a significantly higher percentage of aneuploid blastocysts regardless of the age (12). Retrieving more oocytes at one oocyte retrieval cycle provides more opportunity for euploid blastocysts formation. Despite this, young women with DOR often receive less attention clinically. Our multivariate prediction model offers clinicians more precise and instructive insights. The rationale for focusing on this younger demographic lies in the importance of early identification of potential fertility issues. Recognizing predictive indicators of low oocyte retrieval in this age group can guide discussions on fertility preservation options, crucial for women contemplating future reproductive plans.

It has been reported that women with early-onset DOR potentially compromising oocyte production and quality. However, no significant downstream effects on biological processes appear to impact blastocyst formation (13). Therefore, in this study, we initially utilized a cut-off value of 10 retrieved oocytes as an indicator to predict cumulative live birth. Inge et al. demonstrated that women who yielded low numbers of oocytes utilized an average of 9.6 oocytes per live birth, which was consistent with the results in our study (14). Subsequently, LASSO regression identified significant factors associated with the risk of retrieving fewer than 10 oocytes at one oocyte retrieval cycle in the training group, including age, AFC, AMH, FSH, and FSH/LH ratio. These factors were then employed to develop a predictive model, visualized through a nomogram. The model exhibited good discrimination and calibration, as evidenced by the AUC, C-index, and calibration plots in both the training and validation groups. Thus, our nomogram model for predicting the risk of retrieving fewer than 10 oocytes at one oocyte retrieval cycle in women ≤35 years old demonstrates fair performance and holds promise for clinical use.

In terms of clinical application, we found that the cut-off value of the nomogram to predict the risk of obtaining fewer than 10 oocytes in one oocyte retrieval cycle was 0.455, by which the patients could be categorized into low-risk group and high-risk group. For patients in the low-risk group, standardized COH protocols could be conducted as the likelihood of obtaining ≥10 oocytes at one oocyte retrieval cycle is high. Conversely, patients classified in the high-risk group may require multiple oocyte retrieval cycles to achieve the desired number of oocytes. However, repeated transvaginal ultrasound-guided needle ovarian aspiration, while a common practice, carries increased potential complications such as infection, bleeding, and fibrosis. To address this, pretreatments involving testosterone, dehydroepiandrosterone (DHEA), coenzyme Q10 (CoQ10), and growth hormone are some of the strategies considered before ovarian stimulation to improve oocyte quality, ovarian response, and the number of oocyte retrieved (15–17). Additionally, Zhao et al. have shown that when using a “freeze-all” strategy, the number of oocytes retrieved is an independent positive predictor of cumulative live birth in women under 35 years of age (18). Therefore, on the other hand, our results suggested that the choice of ovarian stimulation protocol for high-risk group patients must consider both efficacy and safety to safely maximize the number of oocytes retrieved. Currently, GnRH-agonist and GnRH-antagonist protocols are widely used for COH, with estimated mean costs per ongoing pregnancy reported at $7595.28 and $8176.76, respectively, in China (19). Progestin-primed ovarian stimulation (PPOS) was reported to have similar clinical outcomes but was medically and economically superior to GnRH-antagonist or GnRH-agonist protocols (20). Nevertheless, due to early exposure to progesterone, fresh embryo transfer is not feasible with PPOS. For women planning consecutive oocyte retrieval cycles to accumulate more oocytes and transferable embryos, PPOS combined with a “freeze-all” strategy could offer a safe, convenient, and cost-effective approach. Further prospective studies focusing on the efficacy of different ovarian stimulation protocols for women classified in the high risk group are warranted to provide more comprehensive guidance for informed decision-making and personalized fertility management.

This study establish a practical model for predicting live birth based on the number of oocytes retrieved at one oocyte retrieval cycle in women ≤35 years of age. The primary strength of this study lies in its real-world example with a large sample size. Additionally, while most screening processes for predictive indicators rely solely on univariate and multivariate analyses, which may be limited in dealing with multicollinearity between variables, we employed LASSO regression analysis to screen indicators and construct a more refined model. Moreover, our external validation confirmed the good accuracy and conformity of the model, alongside its net benefit. A nomogram, offering a personalized and visual model, was developed to provide clinicians with an intuitive and simple tool for practical prediction. Inevitably, several limitations should be taken into consideration. First, this was a retrospective study, and 1167 women with complete oocyte retrieval data were lost to follow-up after embryo transfer, introducing potential selection bias. Second, all data obtained were from a single center. Although the homogeneity of subjects studied and laboratory measurement bias could be well-controlled, evidence from other countries and centers is needed for external validation of this prediction model. Third, we were unable to account for some routine confounders such as alcohol intake and smoking status. Further studies should focus on which ovarian stimulation protocol would be more applicable in women classified in high-risk group according to this nomogram.

In summary, our study constructed a nomogram for predicting live birth based on the risk of retrieving fewer than 10 oocytes at one oocyte retrieval cycle among young women undergoing IVF/ICSI treatment. Five indicators including age, AFC, AMH, FSH, and FSH/LH ratio were incorporated in the nomogram. It exhibited good discrimination and calibration to some extent. This visual and personalized model of ovarian reserve indicators offers clinicians a simple and intuitive tool for the early identification of reproductive outcomes. Notwithstanding that, more prospective and multicentered studies are needed to validate our predictive tool.

## Data Availability

The raw data supporting the conclusions of this article will be made available by the authors, without undue reservation.

## Supplementary material

Supplementary Figure 1 Flow chart of study design.

Supplementary Figure 2
**(A)**. ROC curves for the prediction of a live birth using the number of good-quality embryos. **(B)**. ROC curves for the prediction of obtaining at least two good-quality embryo using the number of oocytes needed. ROC, receiver operating characteristic.

Supplementary Figure 3 Online dynamic nomogram for the risk of retrieving fewer than 10 oocytes at one oocyte retrieval cycle. The usage of the nomogram is illustrated with an assumptive woman as: 30 years old, AFC=7, AMH=1.0ng/ml, FSH=10U/L, FSH/LH ratio=2. The total score added up to 164 for this woman, which represents approximately 0.683 of probability of retrieving fewer than 10 oocytes at one oocyte retrieval cycle.
