# Supplementary figures and images for: A nomogram model to predict the high risk of lower live birth probability in young women undergoing the first IVF-ET cycle: a retrospective study
Source: Front Endocrinol (Lausanne). 2024 Dec 19;15:1401385. doi: 10.3389/fendo.2024.1401385 (PMC11693585; doi:10.3389/fendo.2024.1401385)

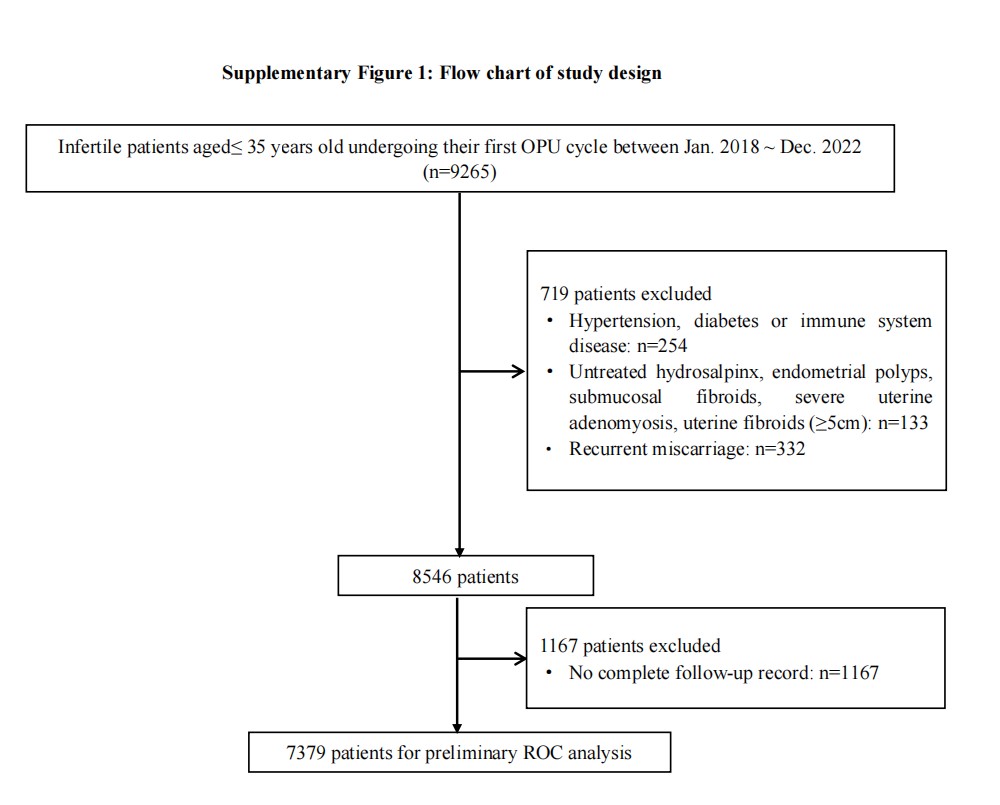

Supplement: Supplementary Figure 1 — Flow chart of study design. [file Image1.jpeg]

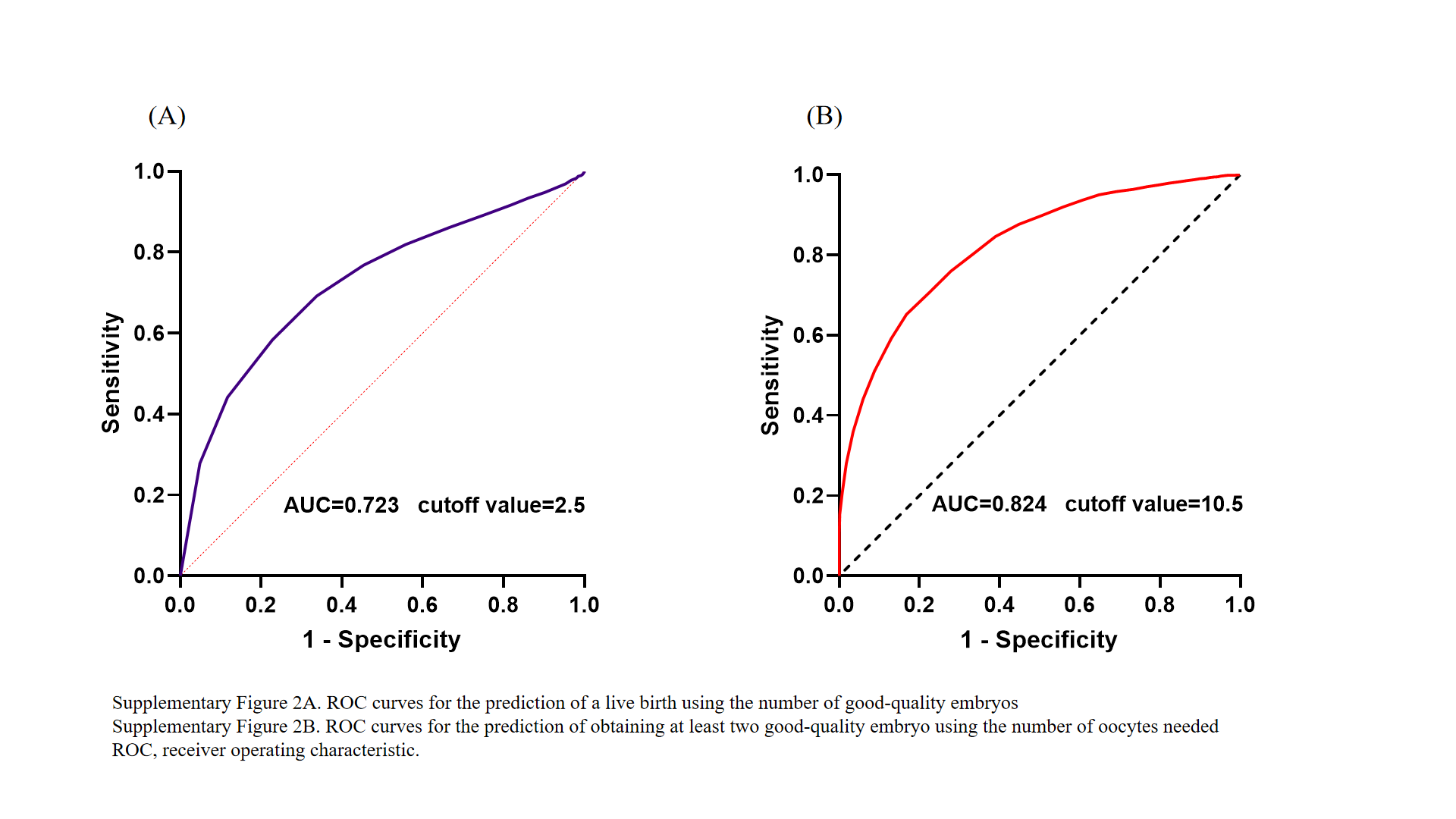

Supplement: Supplementary Figure 2 — (A). ROC curves for the prediction of a live birth using the number of good-quality embryos. (B). ROC curves for the prediction of obtaining at least two good-quality embryo using the number of oocytes needed. ROC, receiver operating characteristic. [file Image2.tif]

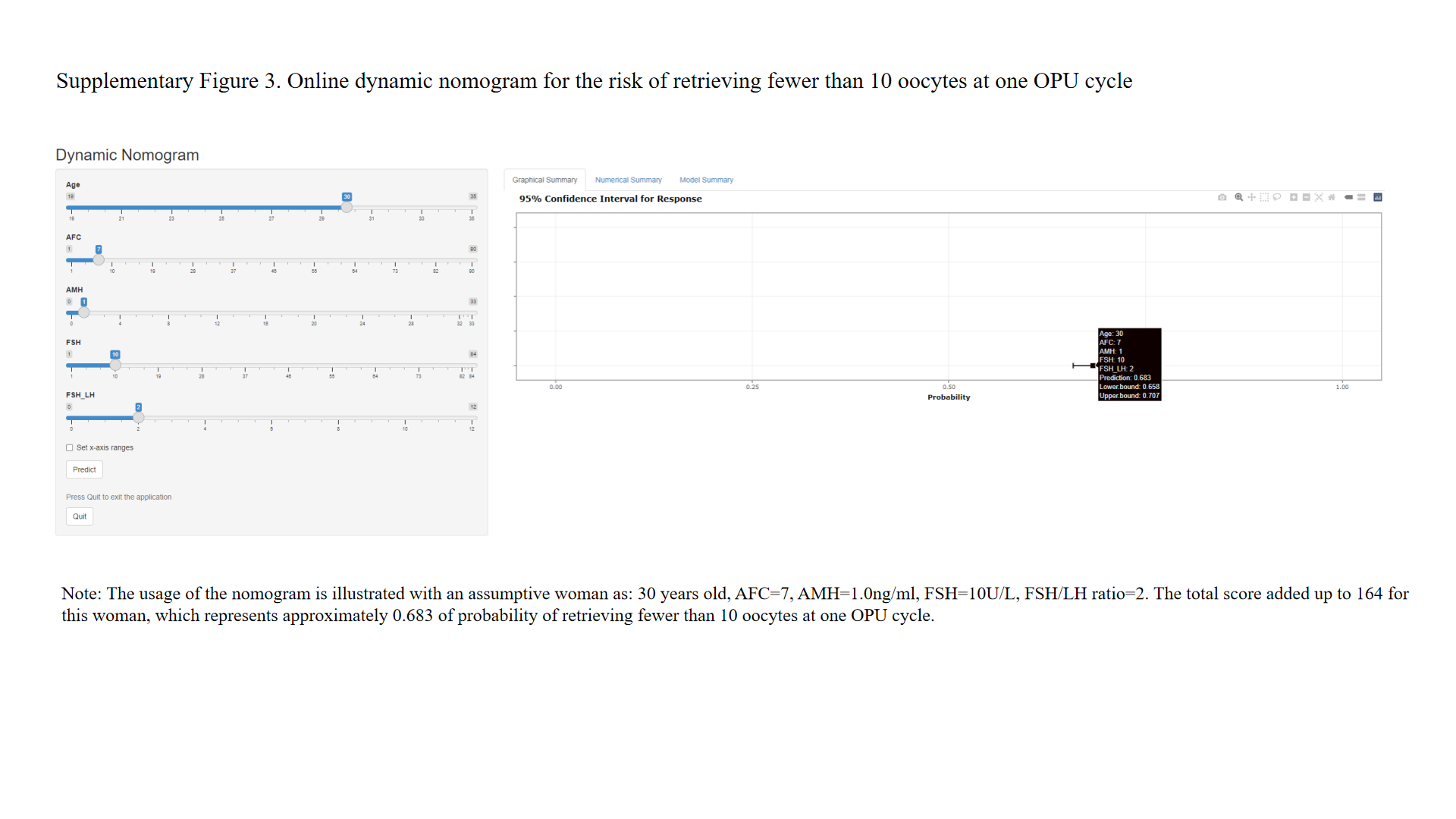

Supplement: Supplementary Figure 3 — Online dynamic nomogram for the risk of retrieving fewer than 10 oocytes at one oocyte retrieval cycle. The usage of the nomogram is illustrated with an assumptive woman as: 30 years old, AFC=7, AMH=1.0ng/ml, FSH=10U/L, FSH/LH ratio=2. The total score added up to 164 for this woman, which represents approximately 0.683 of probability of retrieving fewer than 10 oocytes at one oocyte retrieval cycle. [file Image3.tif]
